# Effect of Sequential Inoculum of Beta-Glucosidase Positive and Probiotic Strains on Brine Fermentation to Obtain Low Salt Sicilian Table Olives

**DOI:** 10.3389/fmicb.2019.00174

**Published:** 2019-02-08

**Authors:** Alessandra Pino, Amanda Vaccalluzzo, Lisa Solieri, Flora V. Romeo, Aldo Todaro, Cinzia Caggia, Francisco Noé Arroyo-López, Joaquin Bautista-Gallego, Cinzia L. Randazzo

**Affiliations:** ^1^Department of Agricultural, Food and Environment, University of Catania, Catania, Italy; ^2^Department of Life Sciences, University of Modena and Reggio Emilia, Reggio Emilia, Italy; ^3^Council for Agricultural Research and Economics, Research Centre for Olive, Citrus and Tree Fruit, Acireale, Italy; ^4^Department of Agricultural, Food and Forest Science, University of Palermo, Palermo, Italy; ^5^Food Biotechnology Department, Instituto de la Grasa, Consejo Superior de Investigaciones Científicas, Universidad Pablo de Olavide, Seville, Spain

**Keywords:** NaCl reduction, microbial debittering, starter cultures, healthy olives, molecular approach

## Abstract

In the present study, the β-glucosidase positive strain *Lactobacillus plantarum* F3. 3 was used as starter during the fermentation of Sicilian table olives (Nocellara Etnea cultivar) at two different salt concentrations (5 and 8%), in order to accelerate the debittering process. The latter was monitored through the increase of hydroxytyrosol compound. In addition, the potential probiotic *Lactobacillus paracasei* N24 strain was added after 60 days of fermentation. Un-inoculated brine samples at 5 and 8% of salt were used as control. The fermentation was monitored till 120 days through physico-chemical and microbiological analyses. In addition, volatile organic compounds and sensorial analyses were performed during the process and at the end of the fermentation, respectively. Lactic acid bacteria and yeasts were, in depth, studied by molecular methods and the occurrence of the potential probiotic N24 strain in the final products was determined. Results highlighted that inoculated brines exhibited a higher acidification and debittering rate than control ones. In addition, inoculated brines at 5% of salt exhibited higher polyphenols (hydoxytyrosol, tyrosol, and verbascoside) content compared to samples at 8% of NaCl, suggesting a stronger oleuropeinolytic activity of the starter at low salt concentration. Lactobacilli and yeasts dominated during the fermentation process, with the highest occurrence of *L. plantarum* and *Wickerhamomyces anomalus*, respectively. Moreover, the potential probiotic *L. paracasei* N24 strain was able to survive in the final product. Hence, the sequential inoculum of beta-glucosidase positive and potential probiotic strains could be proposed as a suitable technology to produce low salt Sicilian table olives.

## Introduction

Among fermented vegetables, table olives are widespread in the Mediterranean area with increasing consumption in both European and non-European countries (International Olive Council, 2016). Olives are intrinsically health thanks to the high content of fiber, vitamins, and polyphenols which play a very important role, exhibiting pharmacological properties and antioxidants effects. In particular, hydroxytyrosol scavenges free radicals, inhibits human low-density lipoprotein (LDL) oxidation, inhibits platelet aggregation, and discloses anticancer activity by means of pro-apoptotic mechanisms (Raederstorff, 2009; Allouche et al., 2011; Buckland and Gonzalez, 2015).

In Sicily, table olives fermentation is mainly performed under traditional methods exploiting the fermentative action of the autochthonous microbiota. Olives are directly brined without previous debittering treatment; therefore, the indigenous microorganisms and the effect of the physico-chemical conditions of brine (pH, salt, presence/absence O_2_, etc.) are mainly responsible of the hydrolysis of the oleuropein and of other β-glucosides. The oleuropein, a β-glucoside compound lending the strong “bitterness” aroma to the olive fruit, is hydrolyzed by β-glucosidases enzymes with the release of glucose and aglycones which are degraded, by an esterase, in the no-bitter phenols hydroxytyrosol, and elenolic acid (Bianchi, 2003). The spontaneous debittering is time consuming and not predictable, and it is strongly influenced by physico-chemical parameters, by the presence of fermentable substrates, and by the autochthonous microbiota. Starter cultures with oleuropein degrading activity were extensively applied in order to reduce the debittering time and to control the fermentation process (Panagou et al., 2003, 2008; Marsilio et al., 2005; Servili et al., 2006; Bevilacqua et al., 2013; Bonatsou et al., 2015), and, among them, the use of β-glucosidase positive strains could be promising (Ghabbour et al., 2011; Tataridou and Kotzekidou, 2015).

Recently, salt intake consumption hypertension and cardiovasular diseases [U.S. Dept. of Agriculture and U.S. Dept. of Health and Human Services, 2010; World Health Organization (WHO), 2012].

Recently, high attention was paid to salt intake since its overmuch intake is considered a risk factor for the onset of hypertension and cardiovascular diseases [U.S. Dept. of Agriculture and U.S. Dept. of Health and Human Services, 2010; World Health Organization (WHO), 2012]. Indeed, the set-up of table olives with low NaCl content is an issue of great interest for the sector (Bautista-Gallego et al., 2013a). However, a complete removal of salt may lead to an increased risk in the survival/ growth of spoilage or food pathogen microorganisms and may also alter food flavor, causing important economic losses. Hence, several studies evaluated the use of KCl, CaCl_2_, and ZnCl_2_ as NaCl replacers. It is well-demonstrated that table olives dealt with NaCl reduction and partial substitution with other salts have a more equilibrated mineral composition, enhancing the consumers' acceptance (Bautista-Gallego et al., 2010, 2011a, 2013b). Nevertheless, the effect of NaCl replaces on sensorial aspects is still controversial and strongly influenced by the concentration of the salt mixture used (Zinno et al., 2017). Recently, Pino et al. (2018a) demonstrated that the reduction of NaCl content to 5%, without any NaCl replacers, did not negatively affect the Nocellara Etnea table olives fermentation, obtaining a microbiologically safe product with appreciate sensorial traits.

Another challenge for vegetable product industry is to satisfy the increasing consumer demand for healthier products. Numerous studies demonstrated that table olives are promising carrier for probiotic strains being able to support their survival, probably thanks to the release of prebiotic substances from fruits. Additionally, their microstructure, in terms of roughness of olive surface, promotes the formation of biofilm that seem to protect probiotic bacteria from stressful conditions (such as acidic environment), favoring their survival through the human gastrointestinal tract (De Bellis et al., 2010; Arroyo-López et al., 2012a; Blana et al., 2014; Randazzo et al., 2017; Rodríguez-Gómez et al., 2017). According to that, the present study was aimed to evaluate the effect of a sequential inoculum of a β-glucosidase positive strain and probiotic bacteria on brine fermentation in order to set up a low salt Sicilian table olives.

## Materials and Methods

### Pilot Scale Olives Processing

Traditionally Sicilian–style table olives from Nocellara Etnea cultivar, provided from a local company, located in Paternò (Sicily), were processed without any lye treatment. Olives were pre-treated as previously reported (Pino et al., 2018a) and directly immersed in sterilized brine, containing 5 or 8% (w/v) of NaCl. The β-glucosidase positive strain *Lactobacillus plantarum* F3.3, previously isolated from fermented table olives was used as starter. The strain was previously characterized for the presence of bglH gene according to Marasco et al. (1998) and its β-glucosidase activity was evaluated by enzymatic assay, according to Sestelo et al. (2004). To set-up probiotic table olives, the potential probiotic *Lactobacillus paracasei* N24, belonging to the Di3A microbial collection was added. This strain was selected according to its technological and probiotic features (Pitino et al., 2010; Randazzo et al., 2010) and for its good ability to survive in table olives (Randazzo et al., 2017; Pino et al., 2018a). Both microorganisms were applied as lyophilized strains.

The experimental fermentation design comprised 8 treatments: 4 fermentations at 5% of NaCl with (F5A; F5B) and without (F5C; F5D) the addition of *L. plantarum* F3.3 strain, and 4 fermentations at 8% of NaCl with (F8A; F8B) and without (F8C; F8D) the addition of *L. plantarum* F3.3. Brine samples F5A; F5B; F8A; F8B were inoculated with the *L. plantarum* starter culture, to a final cell density of 7 log cfu/ml, directly after brining. The potential probiotic *L. paracasei* N24 strain was inoculated in F5B; F5D; F8B; F8D samples after 60 days of brining (at final cell density of 9 log cfu/ml). All fermentations were done at room temperature (18 ± 2°C), and followed up to 120 days. Marine salt was periodically added to maintain the initial concentration and fresh brine was supplied to keep olives totally dipped. Each fermentation was carried out in triplicate.

### Physico-Chemical Analysis

The pH of the brines was detected by using a MettlerDL25 pHmeter (MettlerDL25, Mettler-Toledo International Inc.). Titratable acidity was determined by titring brine samples with 0.1 N NaOH and was expressed as lactic acid (g/100 ml).

The olive brines were filtered through PTFE filters (Millipore, 0.45 μm) and injected in the chromatographic system to analyze the phenol fraction. The HPLC instrument consisted of a chromatography Waters Alliance 2695 HPLC equipped with a Waters 996 photodiode array detector (PDA) set at 280 nm. The column used was a Luna C18 (250 mm × 4.6 mm i.d., 5 μm, 100 Å, Phenomenex, Torrence, CA) which was maintained at 30°C in an oven. The flow rate was 1 mL/min. Separation was obtained by elution gradient using an initial composition of 95% of A solution (2% acetic acid in water) and 5% of B solution (methanol). The concentration of B solution was increased to 30% in 15 min and to 70% in 25 min and then, after 2 min in isocratic condition, the mobile phase was set at the same initial concentration in 8 min. Phenolic compounds were identified by injecting the pure standards of oleuropein, verbascoside, tyrosol, and hydroxytyrosol and by comparing their retention time and UV-Vis spectra. All the analyses were performed in triplicate.

### Microbiological Analyses

Brine samples at 1, 30, 60, 90, and 120 days of fermentation were subjected to microbiological analysis as previously described (Pino et al., 2018a). Mannitol Salt Agar (MSA), incubated at 32°C for 48 h, was used to isolate both coagulase positive and negative staphylococci. All media were provided from Oxoid (Italy) with the exception of YM provided from Difco (Italy). Microbiological analyses were performed in triplicate and results were expressed as log cfu/ml ± standard deviation.

### LAB Isolation and Identification

For each brine sample (F5A, F5B, F5C, F5D, F8A, F8B, F8C, and F8D) and each sampling time (1, 30, 60, 90, and 120 days), 20% of the total number of colonies, recovered on MRS agar plate, were randomly selected, purified, checked for catalase activity and Gram reaction, and microscopically examined before storing in liquid culture using 20% (v/v) glycerol at −80°C. Six-hundred (600) LAB isolates were purified and subjected to total genomic DNA (gDNA) extraction following the method described by Pino et al. (2018b). gDNA concentration and quality were evaluated using the Fluorometer Qubit (Invitrogen, Carlsbad, 278 CA, USA). Multiplex *Rec*A and *Tuf* gene species-specific PCR were performed as previously described (Torriani et al., 2001; Ventura et al., 2003), respectively. Strains not identified at species level with species-specific PCR were subjected to 16S rRNA gene PCR-RFLP analysis according to Pino et al. (2018b). For each PCR-RFLP cluster, the 16S rRNA gene PCR amplicon of one representative strain was purified using the Qiaquick PCR purification kit (Qiagen Hilden, Germany) and was subjected to sequencing and Blast analysis.

### Isolation and Genotypic Identification of Yeasts

From each brine samples at each sampling time, as previously reported, 200 colonies were randomly isolated from YM medium, purified, and microscopically examined prior to storing in liquid culture using 20% (v/v) glycerol at −80°C. For the yeasts characterization, DNA was extracted according to Ruiz-Barba et al. (2005) and subjected to repetitive element palindromic (rep)-PCR analysis by using GTG_5_ primer. The PCR reaction was carried out in a final volume of 25 μl, containing: 5 μl of DNA, 5 μl 5X PCR Buffer, 1 μl of primer GTG_5_ (5-GTGGTGGTGGTGGTG-3), 13.9 μl of filtered water on 0.1 μl of Taq polymerase (Invitrogen, Italy). The amplification program was as follows; an initial denaturation (95°C, 5 min) followed by 30 cycles of denaturation (95°C, 30 s), annealing (40°C, 1 min), and extension (65°C, 8 min) with a single final extension (65°C, 16 min). PCR products were electrophoresed in a 2 % agarose gel in 1X TAE buffer, stained with ethidium bromide (30 min) and visualized under ultraviolet light. The resulting fingerprints were digitally captured and analyzed with the Bionumerics 6.6 software package (Applied Maths, Kortrijk, Belgium). Dendrogram for clustering comparison was built with UPGMA (Unweighted Pair Group Method) method and Pearson correlation.

To validate the clustering analysis and for identification of strains, the 26S rDNA gene of all isolates was further sequenced. The gDNA amplification was performed according to Porrua et al. (2018). PCR products were resolved by electrophoresis on agarose gel (1% w/v) stained with ethidium bromide. DNA ladder plus (Invitrogen, USA) was used to evaluate the molecular weight of amplified DNA. PCR products were purified using Isolate DNA kit (Bioline, USA) according to the manufacturer's instructions and quantified by agarose gel electrophoresis (1% w/v) in 0.5X TBE buffer (89 mM Tris-borate, 2 mM EDTA pH 8). An amount of 10 μl of purified product with forward primer NL1 was used for sequencing by Stab Vida (Lisbon, Portugal). Nucleotide sequences were aligned with the software Molecular Evolution Genetic Analysis (MEGA).

### Rep-PCR for Detecting the Presence of Probiotic *L. paracasei* N24 Strain

Rep-PCR genomic fingerprinting was performed on 79 *L. paracasei* strains, isolated from F5B, F5D, F8B, and F8D brine samples at 120 days of fermentation, using the (GTG)_5_-primer, as described by Versalovic et al. (1994). PCR was carried out in a 20 μl reaction mixture containing 1x Thermo Green buffer (Thermo Scientific, Waltman, MA, USA), 3.0 mM MgCl_2_, 200 μM of each dNTP (Fermentas), 1 U of Taq polymerase (Thermo Scientific, Waltman, MA, USA), 2 μM (GTG)_5_ primer and 50 ng gDNA. Amplifications were performed in a MyCycler thermal cycler (BioRad, Hercules, CA). The PCR cycling parameters and gel running conditions were set according to Solieri et al. (2012). The only modification was the change of annealing temperature from 40 to 45°C. The GeneRuler 100 bp Plus DNA Ladder (Thermo Scientific, Waltman, MA, USA) was used as a molecular size marker. BioDoc Gel Analyzer device (Biometra GmbH, Germany) was used to capture DNA fingerprint images which were then processed through the BioNumerics software v3.0 (Applied Maths, Sint-Martens-Latem, Belgium). Repeatability of rep-PCR was assessed using the inoculated strain N24 as internal control. Pearson's correlation similarity coefficient was chosen to calculate bands patterns similarity matrix with optimization and curve smoothening values at 1%. Unweighted pair group method with arithmetic mean (UPGMA) analysis was exploited to build the (GTG)_5_-based dendrogram.

### Analysis of Volatile Organic Compounds (VOCs)

VOCs analysis was performed on brine samples at 1, 60, and 120 days of fermentation following method and conditions previously described (Randazzo et al., 2017; Pino et al., 2018a) using a gas chromatography-mass-spectrometry (GC-MS). All analyses were performed in triplicate and the results were expressed as means in μg/l of brine.

### Sensory Evaluation of Table Olives

Table olives at 120 days of fermentation were subjected to sensory evaluation by trained panelists (6 females and 4 males, aged from 22 to 40 years). Sensory panel was conducted according to the International Olive Council method (International Olive Council, 2011). Descriptors related to negative sensations, gustatory, and kinaesthetic perceptions were evaluated as previously described (International Olive Council, 2011, 2016; Pino et al., 2018a). In addition, the overall acceptability descriptor, such an indication of the overall quality, was also scored. Sensory data were acquired by a direct computerized registration system (FIZZ Biosystemes. Couternon, France).

### Statistical Analysis

Microbiological and chemical (i.e., single phenol compounds, pH and acidity) data were analyzed by ANOVA (One-way Analysis of Variance) using Tukey's *post-hoc* test, in order to assess the overall differences among treatments. The reference level of significance was 0.01 for chemical assay and 0.05 for VOCs and microbiolgical assays. All statistical analyses were performed using MATLAB software (MathWorks, version 8.5.0), while sensory data were analyzed using the software package Statgraphics® Centurion XVI (Statpoint Technologies, INC.) setting samples as treatments. Data correlations between brine samples differently treated and VOCs were computed using XLStat software (version 2016.1).

## Results

### Physico-Chemical Data

In Table 1, the results of pH and titratable acidity detected in brine samples analyzed thought the fermentation are shown. At the beginning of fermentation, pH values ranged from 5.8 to 6.3, and then they decreased after 30 days. Differences among samples become more appreciable after 60 days of fermentation with the lowest values showed by F5B and F8C samples. At the end of fermentation (120 days) the pH fitted the hygienic limit of 4.3 in all samples (Table 1).

**Table 1:** Results of pH and titratable acidity values in olive brines expressed as means and standard deviations at different time of fermentations.

**Days of fermentation**					
	**1**	**30**	**60**	**90**	**120**
**pH**					
F1	5.9 ± 0.02^ab^	4.6 ± 0.01^bc^	4.4 ± 0.01^bc^	4.4 ± 0.03^c^	4.2 ± 0.03*b*
F2	6.3 ± 0.02^c^	4.5 ± 0.01^ab^	4.3 ± 0.01^ab^	4.2 ± 0.02^a^	4.2 ± 0.01^b^
F3	6.1 ± 0.01^abc^	4.4 ± 0.03^a^	4.4 ± 0.02^bc^	4.3 ± 0.01^b^	4.3 ± 0.03^b^
F4	5.9 ± 0.02^ab^	4.4 ± 0.02^a^	4.4 ± 0.03^bc^	4.2 ± 0.01^a^	4.0 ± 0.02^a^
F5	5.9 ± 0.01^ab^	4.6 ± 0.01^bc^	4.5 ± 0.07^c^	4.4 ± 0.01^c^	4.3 ± 0.03^b^
F6	6.1 ± 0.02^abc^	4.7 ± 0.07^c^	4.5 ± 0.03^c^	4.4 ± 0.03^c^	4.2 ± 0.02^b^
F7	6.2 ± 0.03^bc^	4.5 ± 0.04^ab^	4.2 ± 0.01^a^	4.5 ± 0.03^d^	4.3 ± 0.04^b^
F8	5.8 ± 0.03^a^	4.6 ± 0.04^bc^	4.4 ± 0.01^bc^	4.3 ± 0.02^b^	4.3 ± 0.08^b^
**ACIDITY (g LACTIC ACID 100/ml)**					
F1	0.0225 ± 0.003^a^	0.356 ± 0.001^bc^	0.401 ± 0.001^ab^	0.436 ± 0.015^bc^	0.413 ± 0.016^abc^
F2	0.1010 ± 0.015^b^	0.367 ± 0.017^c^	0.430 ± 0.007^b^	0.526 ± 0.016^d^	0.528 ± 0.013^d^
F3	0.0135 ± 0.002^a^	0.307 ± 0.007^a^	0.368 ± 0.020^a^	0.379 ± 0.032^ab^	0.436 ± 0.015^bc^
F4	0.0082 ± 0.002^a^	0.329 ± 0.008^ab^	0.363 ± 0.003^a^	0.458 ± 0.016^c^	0.458 ± 0.017^c^
F5	0.0133 ± 0.003^a^	0.374 ± 0.008^c^	0.396 ± 0.007^ab^	0.419 ± 0.008^bc^	0.385 ± 0.007^a^
F6	0.0067 ± 0.001^a^	0.385 ± 0.007^c^	0.408 ± 0.08^ab^	0.419 ± 0.008^bc^	0.396 ± 0.007^ab^
F7	0.0082 ± 0.002^a^	0.318 ± 0.008^a^	0.372 ± 0.005^a^	0.352 ± 0.008^a^	0.396 ± 0.008^ab^
F8	0.0077 ± 0.001^a^	0.318 ± 0.008^a^	0.363 ± 0.008^a^	0.396 ± 0.007^abc^	0.420 ± 0.007^abc^

*Data in the same column with different letters are significantly different at P < 0.01*.

The titratable acidity values exhibited an increasing trend in all samples with the exception of F8A and F8B brines, which slightly decreased in acidity at 120 days. The highest acidity values were detected in brines at 5% of NaCl (F5A, F5B, F5C, and F5D). In addition, a significant increase in acidity was recorded after the addition of N24 strain (90 days) mainly in F5B and F5D samples (Table 1).

Results of hydroxytyrosol, tyrosol, oleuropein, and verbascoside quantification are shown in Table 2. Overall, all polyphenols analyzed showed an increasing trend during the fermentation mainly in 5% NaCl brine samples inoculated with starter culture. The highest values of hydroxytyrosol, tyrosol, and verbascoside were mainly recorded in brines inoculated with *L. plantarum* F3.3 starter culture already at 30 days. Similar behavior was observed for oleuropein.

**Table 2:** Results of the analyzed phenols in olive brines expressed as means (mg/l) and standard deviations at different time of fermentations.

**Days of fermentation**					
	**1**	**30**	**60**	**90**	**120**
**HYDROXYTYROSOL**					
F5A	4.77 ± 0.01^c^	104.13 ± 0.78^e^	128.14 ± 0.36^c^	199.81 ± 0.69^c^	267.34 ± 5.56^f^
F5B	5.24 ± 0.11^d^	95.68 ± 0.34^d^	101.38 ± 3.86^a^	166.81 ± 0.79^b^	180.12 ± 0.63^d^
F5C	3.14 ± 0.04^b^	86.86 ± 0.16^c^	112.14 ± 0.23^b^	137.87 ± 1.38^a^	174.32 ± 1.37^cd^
F5D	3.25 ± 0.06^b^	84.93 ± 0.17^bc^	113.47 ± 0.41^b^	151.95 ± 0.49^ab^	160.50 ± 3.87^ab^
F8A	4.97 ± 0.01^c^	94.16 ± 2.28^d^	121.63 ± 1.75^c^	191.93 ± 0.11^c^	192.66 ± 0.30^e^
F8B	4.98 ± 0.02^c^	89.57 ± 1.67^cd^	112.21 ± 1.99^b^	153.63 ± 0.47^ab^	172.98 ± 0.15^cd^
F8C	3.05 ± 0.08^b^	56.89 ± 3.17^a^	112.21 ± 0.49^ab^	142.40 ± 0.47^a^	167.71 ± 2.05^bc^
F8D	2.54 ± 0.07^a^	80.65 ± 0.88^b^	102.57 ± 2.00^a^	147.98 ± 0.69^ab^	152.78 ± 0.90^a^
**TYROSOL**					
F5A	0.0 ± 0.0	8.89 ± 0.01^d^	10.29 ± 0.06^b^	14.77 ± 0.80^e^	19.56 ± 0.16^d^
F5B	0.0 ± 0.0	8.32 ± 0.37^d^	10.28 ± 1.12^b^	14.17 ± 0.09^de^	13.52 ± 0.01^bc^
F5C	0.0 ± 0.0	7.36 ± 0.02^c^	9.37 ± 0.05^ab^	11.29 ± 0.16^a^	13.62 ± 0.14^c^
F5D	0.0 ± 0.0	7.45 ± 0.01^c^	9.66 ± 0.02^ab^	12.58 ± 0.04^bc^	12.66 ± 0.30^abc^
F8A	0.0 ± 0.0	6.79 ± 0.27^bc^	9.35 ± 0.06^ab^	13.56 ± 0.24^cd^	13.51 ± 0.35^bc^
F8B	0.0 ± 0.0	6.62 ± 0.21^b^	8.03 ± 0.36^a^	10.98 ± 0.03^a^	12.08 ± 0.06^a^
F8C	0.0 ± 0.0	4.80 ± 0.06^a^	8.58 ± 0.08*a*	11.29 ± 0.04^a^	12.57 ± 0.45^ab^
F8D	0.0 ± 0.0	6.78 ± 0.04^bc^	8.32 ± 0.05^a^	11.80 ± 0.04^ab^	11.66 ± 0.24^a^
**OLEUROPEIN**					
F5A	0.0 ± 0.0	16.07 ± 2.85^b^	21.97 ± 0.39^e^	35.60 ± 1.61^c^	50.62 ± 0.59^d^
F5B	0.0 ± 0.0	15.41 ± 2.12^b^	11.51 ± 0.71^bc^	20.47 ± 0.93^ab^	21.41 ± 0.18^ab^
F5C	0.0 ± 0.0	9.08 ± 0.22^a^	13.51 ± 0.36^d^	19.86 ± 2.14^ab^	23.43 ± 0.62^abc^
F5D	0.0 ± 0.0	8.30 ± 0.11^a^	12.01 ± 0.33^bc^	15.09 ± 0.45^a^	18.81 ± 1.30^a^
F8A	0.0 ± 0.0	9.06 ± 0.51^a^	12.26 ± 0.05^bcd^	26.99 ± 3.80^b^	28.48 ± 1.22^bc^
F8B	0.0 ± 0.0	8.32 ± 0.18^a^	9.99 ± 0.15^a^	22.38 ± 2.03^ab^	24.64 ± 0.50^abc^
F8C	0.0 ± 0.0	7.55 ± 0.10^a^	12.79 ± 0.03^cd^	19.22 ± 0.56^a^	31.94 ± 3.60^c^
F8D	0.0 ± 0.0	9.13 ± 0.24^a^	11.19 ± 0.01^b^	18.84 ± 1.29^a^	24.02 ± 1.27^abc^
**VERBASCOSIDE**					
F5A	0.0 ± 0.0	31.69 ± 2.39^cd^	32.36 ± 1.95^ab^	50.73 ± 0.17^bc^	65.39 ± 0.41^d^
F5B	0.0 ± 0.0	31.61 ± 1.10^cd^	28.91 ± 3.11^a^	54.53 ± 0.63^cd^	58.57 ± 1.78^c^
F5C	0.0 ± 0.0	30.03 ± 0.21^cd^	31.72 ± 0.06^ab^	33.32 ± 0.42^a^	44.04 ± 0.16^a^
F5D	0.0 ± 0.0	29.41 ± 0.25^bc^	37.59 ± 0.71^bc^	55.40 ± 0.93^de^	52.57 ± 0.68^b^
F8A	0.0 ± 0.0	32.32 ± 0.91^cd^	36.45 ± 3.38^bc^	59.66 ± 0.90^e^	57.66 ± 1.39^c^
F8B	0.0 ± 0.0	33.91 ± 1.27^d^	40.85 ± 1.22^c^	59.75 ± 1.56^e^	56.92 ± 1.75^bc^
F8C	0.0 ± 0.0	18.37 ± 0.40^a^	32.29 ± 0.23^ab^	35.25 ± 2.31^a^	42.53 ± 1.64^a^
F8D	0.0 ± 0.0	25.26 ± 0.41^b^	31.93 ± 0.18^ab^	47.92 ± 0.81^b^	45.75 ± 0.74^a^

*a–f: for each phenols, data in the same column with different superscript letters are significantly different P < 0.01*.

### Microbial Count

Table 3 shown microbial counts of brine samples at both 5 and 8% of NaCl, which is expressed as log cfu/ml. Viable mesophilic bacteria showed different trend among samples. In detail, brines at 5% of NaCl (F5A–F5D), from an initial average value of 7.11 log unit, exhibited a steady trend during the fermentation with slight decrease of cell density after 60 days. Similar behavior was observed for brine samples at 8% of NaCl (F8A–F8D) which showed a mean initial value of 6.73 log unit and a final mean value of 5.56 log unit (Table 3). Regarding LAB population, all brine samples inoculated with starter culture (F5A, F5B, F8A, F8B) presented, at the beginning of fermentation, higher cell density than spontaneous ones (F5C, F5D, F8C, F8D). From 60 to 120 days, LAB reached the highest values in samples inoculated with the potential probiotic N24 strain (F5B, F5D, F8B, F8D). Yeasts were present at an initial level of about 3 log cfu/ml in all experimental brines with the exception of F5A and F5B samples which exhibited initial value of 4.04 and 4.37 log cfu/ml, respectively (Table 3). The yeasts cell densities significantly increased thought the fermentation process, achieving, at 120 days, an average value of 7.37 log unit and 6.64 log unit in brine samples at 5 and 8% of NaCl, respectively. Regarding the staphylococci count, only coagulase negative staphylococci, forming red colonies in the medium, were enumerated and their level, at the beginning of the fermentation, was quite similar among all samples. After a slight increase till 60 days, a decrease to final average values of 3.0 and 3.8 log cfu/ml was achieved in 5 and 8% brine samples, respectively. Similar behavior was observed for enterobacteria counts, which significantly decreased through the fermentation process. At the end of fermentation (120 days) this microbial group was detected, at value below 2 log, in brine samples at 8% of NaCl and below the detection limit in samples at 5% of NaCl, with the exception of the F5D sample. *E. coli* was never detected in any brine samples analyzed (data not shown).

**Table 3:** Microbial counts expressed as log_10_ CFU/ml of 3 replicates ± standard deviation of the main microbial groups detected in experimental brine samples during the fermentation process.

**Microbial groups**	**Days of fermentation**				
	**1**	**30**	**60**	**90**	**120**
**MESOPHILIC BACTERIA**					
F5A	7.34 ± 0.03^f^	7.14 ± 0.03^d^	7.44 ± 0.03^f^	6.79 ± 0.05^e^	5.86 ± 0.04^c^
F5B	7.28 ± 0.02^bd^	6.99 ± 0.10^d^	7.95 ± 0.03^g^	7.43 ± 0.02^f^	6.43 ± 0.01^e^
F5C	6.90 ± 0.07^f^	6.57 ± 0.10^c^	6.35 ± 0.03^d^	5.82 ± 0.03^c^	5.63 ± 0.02^d^
F5D	6.93 ± 0.02^be^	6.44 ± 0.07^c^	6.12 ± 0.06^c^	6.15 ± 0.08^d^	6.94 ± 0.06^f^
F8A	6.72 ± 0.07^acd^	5.84 ± 0.08^ab^	5.76 ± 0.06^b^	5.35 ± 0.03^ab^	4.95 ± 0.03^a^
F8B	6.87 ± 0.03^bc^	5.72 ± 0.03^a^	6.86 ± 0.02^e^	6.94 ± 0.03^e^	6.47 ± 0.09^e^
F8C	6.58 ± 0.10^a^	5.95 ± 0.02^b^	5.38 ± 0.07^a^	5.26 ± 0.05^a^	5.21 ± 0.05^b^
F8D	6.74 ± 0.03^acde^	5.64 ± 0.11^a^	5.51 ± 0.06^a^	5.51 ± 0.08^b^	5.61 ± 0.06^d^
**LACTOBACILLI**					
F5A	7.11 ± 0.07^eg^	7.73 ± 0.11^h^	7.44 ± 0.06^c^	6.92 ± 0.05^d^	6.26 ± 0.13^a^
F5B	7.20 ± 0.06^fg^	7.74 ± 0.11^h^	8.83 ± 0.07^ef^	8.49 ± 0.09^h^	8.29 ± 0.04^c^
F5C	6.09 ± 0.06^a^	6.97 ± 0.06^cfg^	6.78 ± 0.06^b^	6.21 ± 0.42^ac^	6.02 ± 0.11^a^
F5D	6.22 ± 0.06^ab^	6.73 ± 0.08^deg^	8.64 ± 0.10^d^	7.86 ± 0.06^ef^	7.16 ± 0.09^b^
F8A	7.68 ± 0.18^h^	6.69 ± 0.05^bcd^	7.03 ± 0.08^ab^	6.38 ± 0.09^bc^	6.18 ± 0.06^a^
F8B	7.02 ± 0.08^def^	6.74 ± 0.01^bef^	8.14 ± 0.09^fg^	7.98 ± 0.09^fg^	7.10 ± 0.10^b^
F8C	6.59 ± 0.15^bc^	6.25 ± 0.10^a^	6.56 ± 0.06^a^	6.00 ± 0.07^ab^	5.87 ± 0.03^a^
F8D	6.68 ± 0.07^cd^	6.12 ± 0.05^a^	8.58 ± 0.12^eg^	7.63 ± 0.38^eg^	6.94 ± 0.10^b^
**YEASTS**					
F5A	4.04 ± 0.04^de^	4.53 ± 0.05^ce^	5.03 ± 0.04^b^	7.22 ± 0.10^d^	8.39 ± 0.12^c^
F5B	4.37 ± 0.12^e^	4.56 ± 0.10^c^	6.80 ± 0.04^d^	7.14 ± 0.12^d^	7.07 ± 0.13^b^
F5C	3.77 ± 0.10^cd^	4.11 ± 0.14^be^	6.12 ± 0.12^c^	6.42 ± 0.09^c^	7.11 ± 0.14^b^
F5D	3.47 ± 0.10^bc^	6.06 ± 0.08^f^	5.79 ± 0.04^c^	5.55 ± 0.05^a^	6.93 ± 0.09^b^
F8A	3.04 ± 0.04^a^	3.16 ± 0.12^a^	4.37 ± 0.03^a^	5.64 ± 0.03^a^	7.06 ± 0.12^b^
F8B	3.05 ± 0.03^ab^	4.03 ± 0.03^b^	6.13 ± 0.14^c^	6.51 ± 0.03^bc^	6.04 ± 0.04^a^
F8C	3.35 ± 0.14^abc^	4.64 ± 0.04^c^	6.06 ± 0.06^c^	6.65 ± 0.03^bc^	7.22 ± 0.10^b^
F8D	3.24 ± 0.12^ab^	3.42 ± 0.13^a^	4.66 ± 0.13^ab^	5.33 ± 0.03^a^	6.24 ± 0.05^a^
**STAPHYLOCOCCI**					
F5A	4.08 ± 0.07^a^	4.40 ± 0.13^c^	4.62 ± 0.03^a^	4.25 ± 0.11^c^	3.74 ± 0.20^bc^
F5B	4.03 ± 0.09^a^	4.70 ± 0.13^d^	4.80 ± 0.10^ab^	3.42 ± 0.21^ab^	3.04 ± 0.06^a^
F5C	4.15 ± 0.20^a^	4.30 ± 0.24^a^	4.41 ± 0.15^a^	3.68 ± 0.52^ab^	3.21 ± 0.08^a^
F5D	4.06 ± 0.14^a^	4.80 ± 0.05^e^	4.79 ± 0.20^ab^	3.27 ± 0.27^a^	3.07 ± 0.11^a^
F8A	4.85 ± 0.08^b^	5.28 ± 0.22^f^	4.83 ± 0.16^ab^	4.81 ± 0.10^d^	4.28 ± 0.14^d^
F8B	4.21 ± 0.10^a^	4.85 ± 0.07^b^	5.03 ± 0.15^b^	4.77 ± 0.17^d^	3.98 ± 0.03^cd^
F8C	4.80 ± 0.23^b^	5.38 ± 0.10^b^	5.01 ± 0.09^b^	3.76 ± 0.14^b^	3.51 ± 0.29^ab^
F8D	4.31 ± 0.10^a^	4.71 ± 0.24^b^	5.08 ± 0.12^b^	4.47 ± 0.20^cd^	3.48 ± 0.19^ab^
**ENTEROBACTERIA**					
F5A	2.66 ± 0.31^a^	1.19 ± 0.15^a^	<1	<1	<1
F5B	2.29 ± 0.09^a^	1.49 ± 0.31^a^	<1	<1	<1
F5C	2.28 ± 0.17^a^	1.36 ± 0.41^a^	<1	<1	<1
F5D	4.01 ± 0.11^cd^	3.38 ± 0.29^c^	2.18 ± 0.09^a^	1.63 ± 0.15^a^	1.55 ± 0.19^a^
F8A	3.31 ± 0.21^b^	3.06 ± 0.10^bc^	2.16 ± 0.15^a^	1.38 ± 0.27^a^	1.21 ± 0.15^a^
F8B	3.88 ± 0.16^bc^	3.32 ± 0.19^bc^	2.57 ± 0.23^a^	1.14 ± 0.10^a^	1.63 ± 0.14^a^
F8C	3.41 ± 0.15^bd^	2.72 ± 0.39^b^	2.00 ± 0.18^a^	1.31 ± 0.17^a^	1.27 ± 0.17^a^
F8D	3.43 ± 0.11^bd^	2.75 ± 0.11^b^	2.04 ± 0.06^a^	1.28 ± 0.20^a^	1.24 ± 0.14^a^

*a–h: for each medium, data in the same column with different superscript letters are significantly different (P < 0.05)*.

### Molecular Identification of LAB

**Figure 1:**
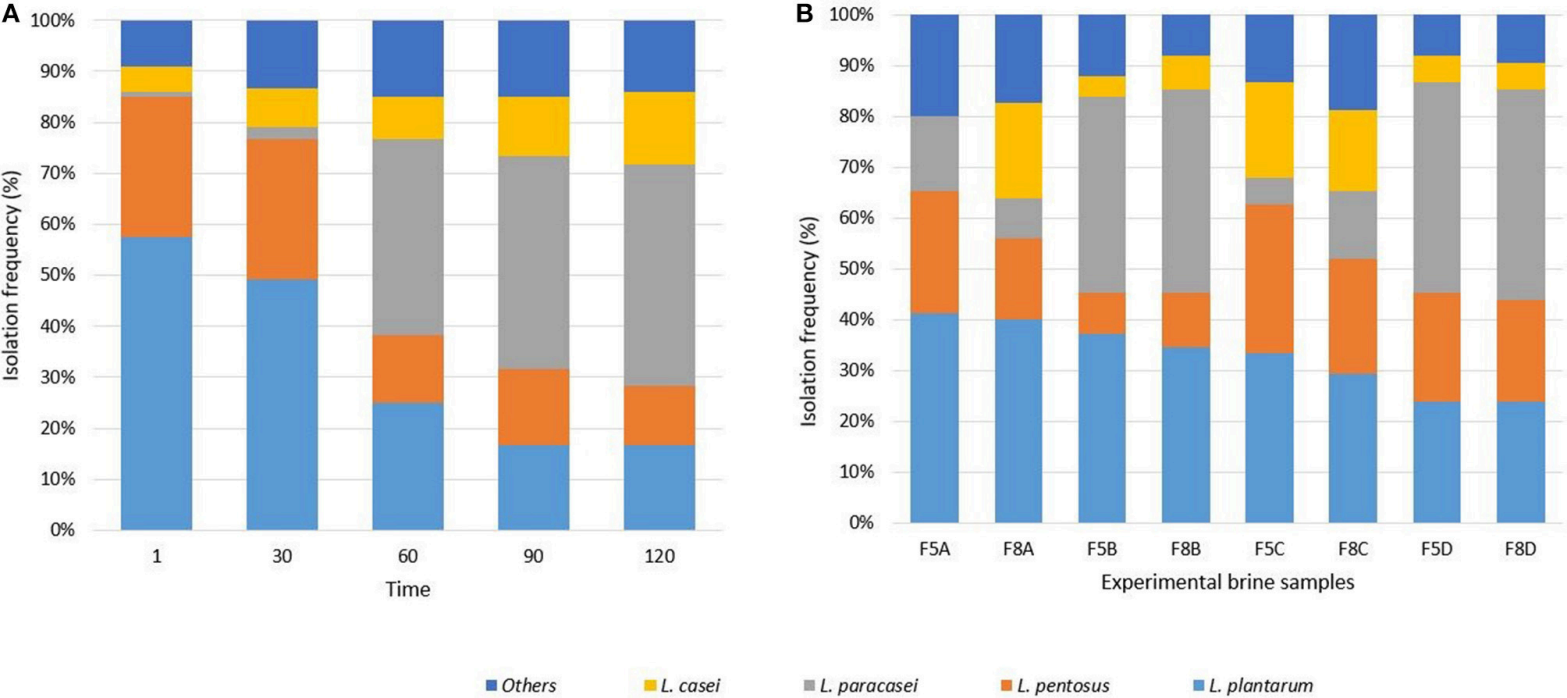
Isolation frequency, expressed in percentage, of LAB species throughout the fermentation **(A)** and in the experimental brine samples **(B)**.

Six hundred isolates from MRS agar plates were considered LAB based on their positive Gram reaction, non-motility, absence of catalase activity and spore formation, as well as rod or coccal shape. R*ec*A and *Tuf* gene species-specific PCRs revealed the presence of strains belonging to *L. plantarum, Lactobacillus pentosus, L. paracasei*, and *Lactobacillus casei* species. The distribution of lactobacilli at different sampling times is reported in Figure 1A and their occurrence in the different brine samples is showed in Figure 1B. Isolates not identified at species level were indicated as “others.” Evaluating the distribution of LAB strains through the fermentation, results indicated that *L. plantarum* and *L. pentosus* represented the dominant species at the beginning of the process (till 30th days) and a high occurrence of *L. paracasei* species was detected up to 60 days. Whereas, the highest occurrence of *L. casei* strains was achieved at 120 days (Figure 1A). Zooming on the fermentation at different salt content (5 and 8%) differently inoculated, results showed the dominance of *L. plantarum* strains in samples inoculated with starter, the highest occurrence of *L. casei* and *L. pentosus* in spontaneous samples (F5C, F8C, F5D, F8D) and of *L. paracasei* in samples inoculated with the probiotic strain N24 (Figure 1B).

Isolates not identified initially with *rec*A and *Tuf* gene primer pairs were subjected to PCR-RFLP analysis of the 16SrDNA and clustered into four different groups (data not shown). One representative isolate for each cluster was identified by 16S rRNA gene sequencing and were deposited in the GenBank database. The species attribution and the accession numbers of the sequenced strains were as follows (isolates code in parentheses): *Leuconostoc mesenteroides* MK085109 (F5C.1), *Lactococcus lactis* MK085110 (F5B.38), *Lactobacillus brevis* MK085111 (F5D.44), and *Enterococcus faecium* MK085112 (F5A.21).

### Molecular Identification of Yeasts

**Figure 2:**
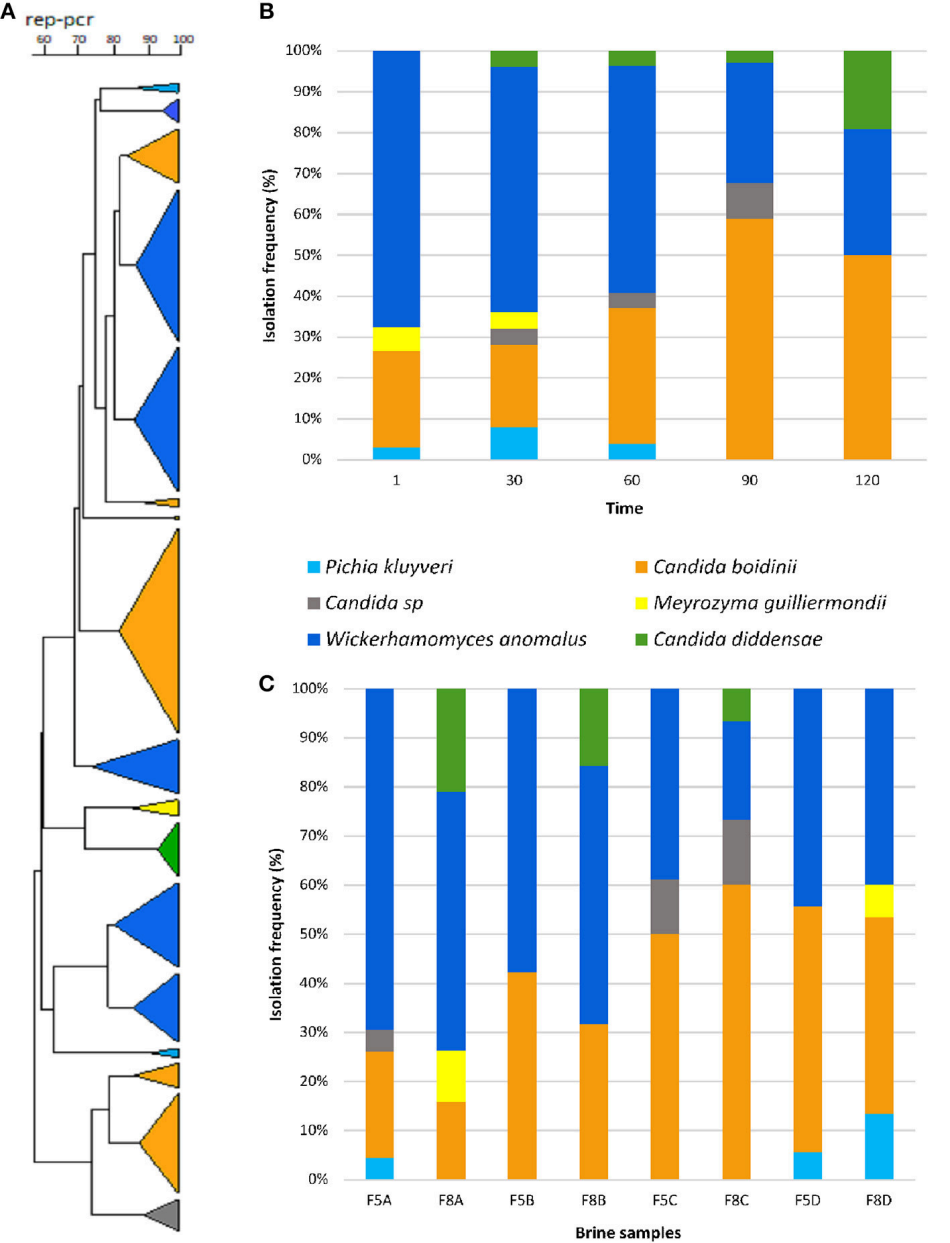
Dendrogram generated after bioinformatics analysis with Bionumerics 6.6 software package of the rep-PCR profiles obtained with GTG_5_ **(A)**. Isolation frequency (%) of yeasts during the fermentation time of Nocellara Etnea table olives **(B)**. Distribution of yeast species in different experimental brine samples **(C)**.

Two-hundred yeast isolates were randomly obtained during the fermentation process. The dendogram generated by rep-PCR with primer GTG_5_ showed that the isolates formed 17 groups clearly differentiated. The most numerous groups belonged to the *Wickerhamomyces anomalus* and *Candida boidinii* species, although representatives of *Candida diddensiae, Pichia kluyveri* and *Meyerozyma guillermondii* were also identified (Figure 2A).

The evolution of the different yeast species throughout the fermentative process is presented in Figure 2B. At this regard, *W. anomalus* and *C. boidinii* formed a stable dual species consortium through the fermentation, since they were both detected more frequently than others species, with a mean frequency of 49 and 37%, respectively. Indeed, these species were dominant in all brine samples differently treated (Figure 2C). The rest of the species were isolated at very low mean frequencies; in particular, *M. guillermondii* (1.97%) was detected only in samples at 8% of NaCl till 30th days of fermentation, whereas, *P. kluyveri* (2.98%) in samples F5A, F5D, and F8D till 60 days and *C. diddensiae* (5.87%) was detected starting from the 30th day of fermentation only in brines at 8% of NaCl (Figure 2C).

### Detection of *L. paracasei* N24 Strain at 120 Days of Fermentation

**Figure 3:**
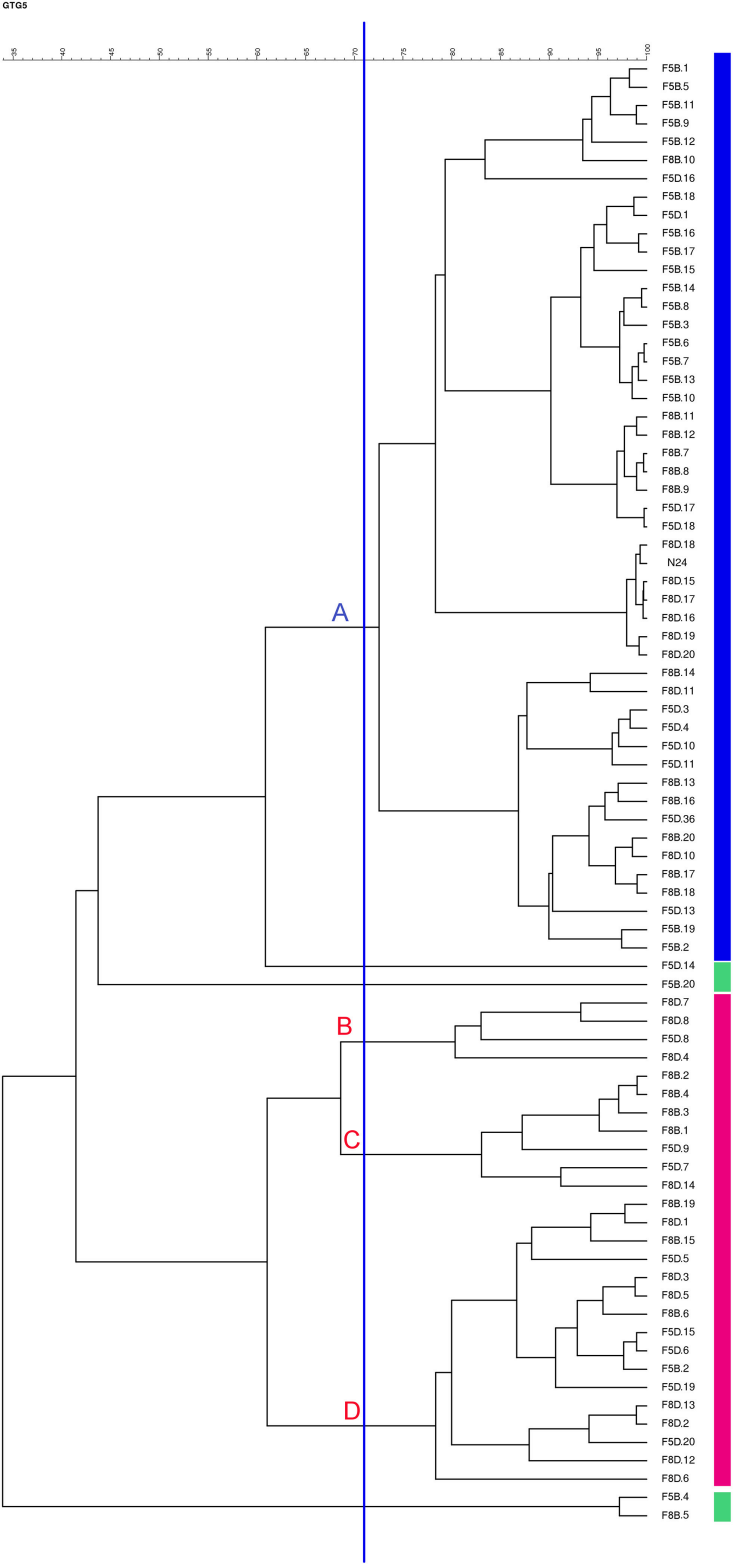
Dendrogram generated after cluster analysis of the digitized GTG_5_-PCR fingerprints of the *Lactobacillus paracasei* strains isolated from F5B, F5D, F8B, and F8D brine samples at 120 days of fermentation.

The presence of inoculated strain N24 was assessed at 120 days of fermentation by rep-PCR with primer GTG_5_ on a pool of 79 *L. paracasei* isolates from samples F5B, F5D, F8B, and F8D. Preliminarily, the rep-PCR repeatability was evaluated using gDNA from strain N24 as internal control in four different gels, obtaining a similarity of 74.3% (data not shown). Accordingly, this value was retained as similarity threshold to establish the identity of isolates compared to the rep-PCR profile of promising probiotic strain N24. The dendrogram generated using the GTG_5_-based patterns of *L. paracasei* isolates revealed the presence of four major clusters (from A to D) and four singleton *L. paracasei* isolates below 74.3% similarity (Figure 3; Supplementary Figure 1). The cluster analysis showed that the major cluster A grouped both the inoculated strain N24 and 48 out of 79 isolates, indicating that these isolates were assimilated to the N24 strain profile. The majority of them were isolated from samples F5B and F8B, over the indigenous *L. paracasei* isolates. In detail, out of 48, 18 strains were isolated from F5B, 12 from F8B, 10 from F5D, and 8 from F8D. The remaining isolates belonged to clusters B, C, and D.

### Volatile Organic Compounds (VOCs)

Results of VOCs of different brine samples at 60 and 120 days of fermentation are reported in Table 4. Overall, 46 compounds as acids, alcohols, esters, aldehydes, and phenols were identified, exhibiting a growing trend through the fermentation, with the exception of samples F5A, F5D, F8A, and F8D. The highest value was registered in samples F8C, with a value of 2,739.17 μg/l. Alcohols were the most abundant compounds, followed by esters and acids, whereas aldehydes and phenols were detected at lower concentrations. Focusing on each compound, among alcohols, ethanol dominated the fermentation process showing an increase only in spontaneous samples (F5C and F8C), followed by isoamylalcohol and phenylethylalcohol, which registered a variable trend through the fermentation (Table 4). Ethyl-acetate and methyl 2-methylbutanoate were the main detected esters. Among acids, the acetic acid was the most abundant compound, with the highest value in F5C and F8C samples. The most abundant aldehydes and phenols were nonanal, benzaldehyde and creasol, respectively (Table 4).

**Table 4:** Volatile organic compounds (VOCs) expressed as μg/l of experimental brine samples at 60 and 120 days of fermentation.

**Compounds**	**rt**	**T0**	**F5A 60**	**F5A 120**	**F5B 60**	**F5B 120**	**F5C 60**	**F5C 120**	**F5D 60**	**F5D 120**	**F8A 60**	**F8A 120**	**F8B 60**	**F8B 120**	**F8C 60**	**F8C 120**	**F8D 60**	**F8D 120**
**Acids**		**0.00**	**48.46**	**92.24**	**70.48**	**150.75**	**74.29**	**124.08**	**49.57**	**40.69**	**57.77**	**35.47**	**81.06**	**111.28**	**58.14**	**135.11**	**35.60**	**50.30**
Acetic acid	24.78	0.00^a^	26.43^b^	58.74^e^	46.26^d^	77.75^f^	72.30^f^	96.15^g^	44.02^d^	39.67^c^	29.76^b^	35.47^c^	50.02^de^	62.01^e^	58.14^e^	106.25^h^	35.60^c^	34.45^c^
Propionic acid	29.74	0.00^a^	4.15^d^	3.62^d^	5.22^e^	32.50^f^	1.01^b^	2.31^c^	0.00^a^	1.02^b^	5.84^e^	0.00^a^	2.06^c^	0.65^b^	0.00^ha^	3.14^d^	0.00^a^	0.96^b^
Isobutyric acid	31.43	0.00^a^	0.91^b^	0.25^b^	1.28^bc^	3.10^c^	0.98^b^	1.98^c^	0.00^a^	0.00^a^	1.02^b^	0.00^a^	6.56^d^	2.01^c^	0.00^a^	2.26^c^	0.00^a^	0.00^a^
Butanoic acid	35.01	0.00^a^	0.32^b^	0.62^b^	0.90^b^	4.37^c^	0.00^a^	0.15^b^	5.55^c^	0.00^a^	0.62^b^	0.00^a^	1.05^b^	10.60^d^	0.00^a^	0.96^b^	0.00^a^	0.00^a^
Hexanoic acid	36.19	0.00^a^	8.96^b^	27.89^f^	7.61^b^	31.99^f^	0.00^a^	13.67^cd^	0.00^a^	0.00^a^	10.54^c^	0.00^a^	18.21^e^	34.12^f^	0.00^a^	12.63^c^	0.00^a^	14.89^a^
2-Ethyleptanoic acid	57.20	0.00^a^	7.69^d^	1.12^b^	9.21^e^	1.04^b^	0.00^a^	9.82^e^	0.00^a^	0.00^a^	9.99^e^	0.00^a^	3.16^c^	1.89^b^	0.00^a^	9.87^e^	0.00^a^	0.00
**Alcohols**		**63.03**	**519.08**	**287.57**	**494.50**	**437.32**	**914.38**	**1662.74**	**276.30**	**152.56**	**539.05**	**312.90**	**962.57**	**995.99**	**960.57**	**1842.36**	**229.37**	**191.88**
Ethanol	3.33	6.77^a^	290.25^c^	151.13^b^	315.24^c^	162.54^b^	582.12^d^	1325.14^e^	164.48^b^	83.96^b^	305.17^c^	164.48^b^	639.44^d^	669.21^d^	615.98^d^	1485.32^e^	152.36^b^	125.87^b^
Isoamylalcohol	11.58	0.00^a^	79.84^d^	2.61^a^	48.91^c^	69.71^d^	78.83^d^	118.13^e^	22.14^b^	20.16^b^	83.78^d^	3.01^a^	75.12^d^	78.65^d^	78.01^d^	129.58^e^	1.03^a^	23.04^b^
1-Hexanol	19.23	0.00^a^	9.52^bc^	8.44^b^	12.47^c^	15.86^d^	13.15^c^	20.14^e^	13.52^c^	11.33^c^	10.03^c^	7.55^b^	18.39^e^	15.19^d^	13.84^c^	21.06^e^	3.69^a^	3.52^a^
cis Hexen 1 ol	20.84	0.00^a^	21.01^d^	22.63^d^	29.82^e^	28.56^e^	59.62^g^	83.22^i^	14.25^c^	7.86^b^	22.97^d^	31.24^e^	71.98^h^	52.12^f^	61.24^g^	89.61^i^	5.12^b^	1.14^a^
3-Octenol	25.13	0.00^a^	0.00^a^	0.96^a^	0.86^a^	1.99^b^	2.05^b^	2.63^bc^	0.00^a^	0.00^a^	0.00^a^	0.62^a^	3.92^d^	1.84^b^	1.16^a^	2.98^c^	0.00^a^	0.00^a^
1-Eptanol	25.79	2.98^b^	3.10^b^	3.54^b^	5.01^c^	5.03^c^	12.13^e^	5.14^c^	1.07^a^	0.93^a^	2.44^b^	2.58^b^	8.52^d^	3.01^b^	9.84^d^	6.23^c^	0.00^a^	0.00^a^
1-Octanol	31.12	19.58^f^	68.11^i^	34.99^h^	2.84^b^	11.84^d^	23.14^g^	3.21^c^	0.86^a^	0.51^a^	65.28^i^	37.37^h^	9.68^c^	8.72^c^	23.69^g^	3.02^b^	14.32^e^	0.00^a^
1-Nonanol	35.91	4.11^c^	0.00^a^	1.05^b^	0.00^a^	0.00^a^	23.96^d^	0.00^a^	0.00^a^	0.00^a^	0.00^a^	0.00^a^	0.00^a^	0.00^a^	22.10^d^	0.00^a^	0.00^a^	0.00^a^
Benzyl alcohol	47.77	0.00^a^	7.42^c^	14.26^d^	9.63^c^	13.65^d^	13.68^d^	1.01^a^	4.42^b^	3.63^b^	8.12^c^	9.08^c^	29.63^g^	22.13^f^	16.54^e^	0.00^a^	0.00^a^	0.00
Phenylethyl alcohol	50.96	0.00^a^	39.83^c^	47.96^cd^	69.72^d^	128.14^ef^	102.56^e^	103.89^e^	55.56^d^	24.18^b^	41.26^c^	56.97^d^	105.89^e^	145.12^f^	114.21^e^	104.56^e^	52.85^d^	38.31^a^
1-U < 1ecanol	53.54	27.49^c^	0.00^a^	0.00^a^	0.00^a^	0.00^a^	3.14^b^	0.23^a^	0.00^a^	0.00^a^	0.00^a^	0.00^a^	0.00^a^	0.00^a^	3.96^b^	0.00^a^	0.00^a^	0.00^a^
**Esters**		**4.09**	**181.46**	**200.51**	**283.60**	**421.49**	**334.35**	**448.16**	**284.96**	**363.63**	**274.22**	**358.87**	**287.75**	**433.17**	**378.62**	**579.82**	**306.71**	**308.86**
Ethyl acetate	2.75	0.00^a^	140.11^b^	145.91^b^	129.71^b^	149.52^b^	147.22^b^	248.78^d^	136.82^b^	298.72^e^	205.62^c^	244.42^d^	132.71^b^	178.12^c^	205.61^c^	325.16^e^	222.53	195.93
Ethyl propanoate	3.56	0.00^a^	5.71^b^	1.49^a^	0.00^a^	7.02^b^	0.00^a^	0.00^a^	0.00^a^	0.00^a^	7.69^b^	7.71^b^	0.00^a^	0.00^a^	0.00^a^	0.00^a^	0.00^a^	0.00^a^
Ethyl butanoate	4.07	0.00^a^	0.00^a^	2.50^a^	1.88^a^	9.02^c^	3.96^b^	1.02^a^	0.00^a^	0.00^a^	7.53^c^	0.00^a^	2.48^a^	10.86^c^	8.88^c^	0.00^a^	0.00^a^	0.00^a^
Methyl 2-methylbutanoate	5.44	0.00^a^	0.00^a^	8.37^b^	15.12^c^	91.13^g^	12.33^c^	61.20^e^	11.72^c^	0.00^a^	0.00^a^	22.94^d^	6.42^b^	83.25^g^	25.84^d^	70.58^f^	10.83^bc^	14.18^c^
Methyl 3-methylbutanoate	5.85	0.00^a^	0.00^a^	0.72^a^	12.09^e^	61.05^g^	1.04^a^	8.52^d^	0.00^a^	1.14^a^	0.00^a^	16.24^f^	6.23^c^	16.14^f^	2.51^b^	10.58^e^	0.00^a^	15.42^f^
Isoamylacetate	7.47	0.00^a^	8.52^c^	0.44^a^	8.13^c^	10.28^c^	105.31^l^	39.18^f^	96.21^i^	25.47^e^	16.18^d^	38.55^f^	5.62^b^	18.12^d^	3.46^b^	44.01^g^	54.99^h^	57.92^h^
Methyl hexanoate	9.90	0.00^a^	0.00^a^	2.43^a^	0.00^a^	0.00^a^	0.00^a^	0.21^a^	0.00^a^	0.47^a^	14.83^b^	0.00^a^	0.00^a^	0.00^a^	0.00^a^	0.00^a^	0.00^a^	0.00^a^
Ethyl hexanoate	12.29	0.00^a^	0.00^a^	0.46^a^	7.58^c^	4.02^b^	3.65^b^	3.68^b^	0.00^a^	0.00^a^	0.00^a^	0.00^a^	11.13^e^	9.06^d^	9.01^d^	5.12^b^	0.00^a^	0.00^a^
Ethyl lactate	18.49	0.00^a^	0.00^a^	6.14^b^	29.34^e^	39.42^f^	10.97^c^	31.74^e^	0.00^a^	5.11^b^	0.00^a^	0.00^a^	32.67^e^	52.36^h^	23.12^d^	45.49^g^	0.00^a^	0.00^a^
Methyl 2-hydroxy-3-methylbutanoate	23.10	0.00^a^	0.00^a^	0.00^a^	0.00^a^	3.96^b^	0.21^a^	7.76^c^	0.00^a^	0.00^a^	0.00^a^	11.09^d^	0.00^a^	4.05^b^	0.00^a^	8.23^c^	0.00^a^	0.00^a^
Ethyl octanoate	23.44	0.00^a^	0.00^a^	2.95^c^	1.87^b^	0.26^a^	1.48^b^	1.02^b^	0.00^a^	4.60^c^	0.00^a^	0.00^a^	3.02^c^	0.75^b^	9.09^d^	1.81^b^	0.00^a^	0.00^a^
Ethyl-3-hydroxybutyrate	28.92	0.00^a^	18.04^d^	9.20^c^	12.47^c^	6.99^b^	0.00^a^	0.80^a^	0.00^a^	8.17^b^	0.00^a^	0.00^a^	3.82^b^	6.31^b^	0.00^a^	1.63^b^	0.00^a^	10.85^c^
Ethyl 2-hydroxy-4-methylpentanoate	30.00	0.00^a^	1.50^a^	10.49^e^	6.15^c^	7.54^d^	3.96^b^	7.14^d^	9.38^e^	4.67^c^	3.60^b^	5.06^c^	11.36^f^	12.12^f^	6.77^cd^	14.75^g^	3.54^b^	5.60^c^
Methyl decanoate	32.95	2.95^b^	0.00^a^	0.00^a^	0.00^a^	0.00^a^	0.00^a^	0.00^a^	0.00^a^	0.00^a^	0.00^a^	0.00^a^	0.00^a^	0.00^a^	0.00^a^	0.23^a^	0.00^a^	0.00^a^
Ethyl decanoate	35.77	0.00^a^	0.00^a^	0.00^a^	1.24^b^	0.33^a^	1.97^b^	2.23^b^	0.00^a^	0.00^a^	0.00^a^	0.00^a^	1.68^b^	1.46^b^	1.86^b^	4.96^c^	0.00^a^	0.00^a^
Ethyl benzoate	36.74	0.00^a^	0.00^a^	0.95^b^	13.82^d^	3.68^c^	1.45^b^	1.88^b^	0.00^a^	0.00^a^	0.00^a^	0.00^a^	14.58^d^	5.02^c^	2.41^b^	5.12^c^	0.00^a^	0.00^a^
Acetic acid, 2 phenylethyl	43.01	0.00^a^	0.00^a^	1.68^b^	7.28^d^	2.13^b^	0.68^a^	1.63^b^	0.00^a^	0.00^a^	0.00^a^	0.00^a^	1.73^b^	2.36^b^	1.72^b^	4.16^c^	0.00^a^	0.00^a^
Methyl hydrocinnamate	45.91	1.14^b^	0.00^a^	3.65^c^	0.00^a^	7.61^e^	5.23^d^	2.22^b^	0.00^a^	0.00^a^	0.00^a^	0.00^a^	10.03^f^	12.13^f^	10.36^f^	5.16^d^	0.00^a^	0.00^a^
Ethyl dodecanoate	46.20	0.00^a^	0.00^a^	0.00^a^	8.36^c^	0.48^a^	0.71^a^	9.04^c^	6.03^b^	0.00^a^	8.55^c^	0.00^a^	12.12^d^	1.58^a^	1.12^a^	21.85	0.00	0.00
Ethyl hydrocinnamate	48.97	0.00^a^	7.58^c^	3.12^b^	28.56^l^	17.05^g^	34.18^m^	20.11^h^	24.80^i^	15.28^f^	10.22^cd^	12.86^e^	32.15^m^	19.48^h^	66.86^n^	10.98^d^	14.82^f^	8.96^c^
**Aldheydes**		**149.90**	**87.40**	**62.52**	**36.83**	**72.14**	**44.29**	**50.71**	**32.71**	**1.40**	**120.28**	**117.41**	**53.09**	**34.44**	**42.84**	**71.32**	**45.34**	**16.55**
Octanal	14.81	14.12^e^	10.58^d^	6.70^c^	6.52^c^	5.98^bc^	6.48^c^	5.12^b^	0.00^a^	0.61^a^	9.21^d^	10.64^d^	13.58^e^	5.86^b^	4.47^b^	9.36^d^	0.00^a^	0.00^a^
Nonanal	20.78	65.32^l^	40.78^h^	4.25^b^	11.93^d^	31.85^g^	14.56^d^	18.17^e^	13.25^d^	0.00^a^	32.76^g^	52.07^i^	19.63^e^	9.86^c^	14.02^d^	25.12^f^	24.46^f^	1.76^b^
3-Octanal	21.64	0.00^a^	0.00^a^	1.90^b^	1.58^b^	1.96^b^	2.36^b^	2.36^b^	1.74^b^	0.79^a^	0.00^a^	26.47^e^	2.56^b^	1.02^a^	2.36^b^	2.15^b^	12.78^d^	5.27^c^
Decanal	26.85	68.82^h^	30.75^f^	41.89^g^	6.78^b^	18.44^d^	9.57^c^	9.99^a^	7.46^b^	0.00^a^	24.09^e^	0.00^a^	5.69^b^	5.68^b^	11.00^c^	18.96^d^	0.00^a^	7.47^b^
Benzaldehyde	28.08	1.64^b^	5.29^c^	7.78^d^	10.02^e^	13.91^f^	11.32^e^	15.07^f^	10.26^e^	0.00^a^	54.22^h^	28.23^g^	11.63^e^	12.02^e^	10.99^e^	15.73^f^	8.10^d^	2.05^b^
**Phenols**		**0.00**	**102.59**	**49.90**	**21.94**	**144.69**	**116.02**	**146.22**	**26.95**	**72.45**	**137.38**	**147.21**	**7.80**	**43.19**	**112.46**	**110.56**	**5.61**	**2.24**
Guaiacol	47.25	0.00^a^	0.00^a^	2.99^b^	0.00^a^	29.08^d^	57.16^e^	61.12^f^	12.85^c^	3.58^b^	0.00^a^	0.00^a^	0.00^a^	11.02^c^	56.23^e^	53.01^e^	0.00^a^	0.00^a^
Creosol	52.57	0.00^a^	102.59^h^	43.69^e^	10.50^b^	98.65^g^	17.89^bc^	44.32^e^	12.54^a^	52.36	95.11	106.62	3.16	23.14	19.67	28.15	0.00	0.00
Phenol	55.36	0.00^a^	0.00^a^	0.63^a^	1.33^b^	3.56^c^	5.69^d^	9.75^e^	1.56^b^	2.58^c^	0.00^a^	0.00^a^	0.23^a^	1.02^b^	4.50^d^	10.42^e^	0.00^a^	0.00^a^
4-Ethyl phenol	63.20	0.00^a^	0.00^a^	2.59^b^	10.11^d^	13.40^e^	35.28^g^	31.03^h^	0.00^a^	13.93^e^	42.27^h^	40.59^h^	4.41^c^	8.01^d^	32.06^g^	18.98^f^	5.61^c^	2.24^b^
Total		217.02	938.99	692.74	907.35	1226.39	1483.33	2431.91	670.49	630.72	1128.70	971.86	1392.27	1618.07	1552.63	2739.17	622.63	569.83

*a–h: for each compound different superscript letters in the same raw are significantly different (P < 0.05). Bold value indicates VOCs class and the total of them*.

**Figure 4:**
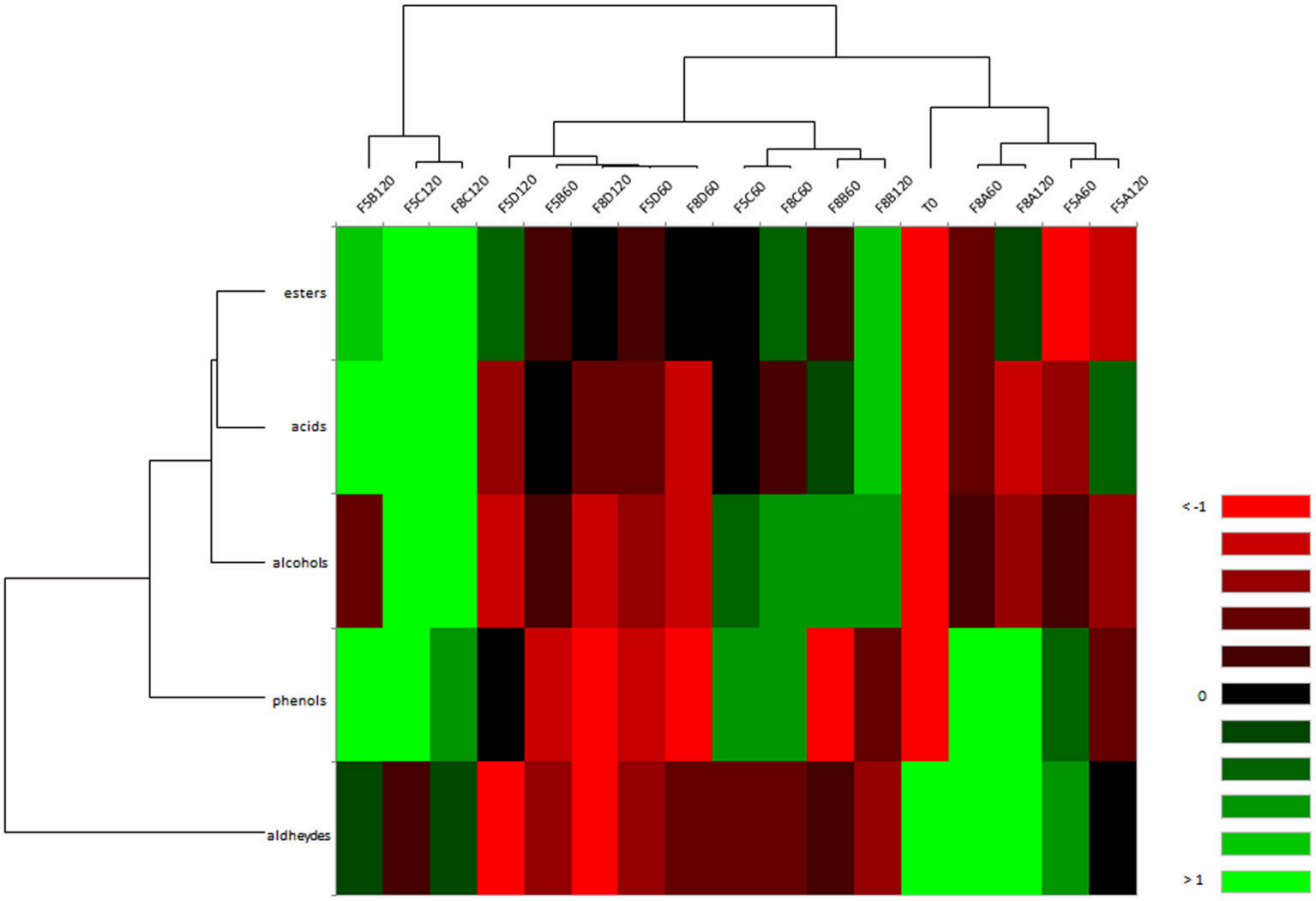
Heat map of correlation between VOCs and F5 (A-D) and F8 (A-D) brine samples at 60 and 120 days of fermentation. The colors of the scale bar denote the nature of the correlation, with 1 indicating a perfectly positive correlation (green) and −1 indicating a perfectly negative correlation (red) between VOCs and brine samples.

Figure 4 shows correlation between VOCs and brine samples differently treated. Overall, it is possible to point out that the salt concentration did not influence the VOCs formation through fermentation in brine samples, which were mainly grouped based on the treatment (starter and/or probiotic addition and spontaneous). In detail, samples inoculated with starter (F5A and F8A) were clustered together, showing a negative correlation with alcohol and ester compounds; spontaneous brine samples inoculated with the probiotic strain N24 (F5D and F8D) were negatively correlated to phenols, aldehydes and alcohols. Different correlations were detected for samples inoculated with both starter and probiotic strains (F5B and F8B). In particular, sample F8B at 60 and 120 days of fermentation were grouped together, exhibiting a positive correlation with alcohols and acids and a negative correlation with phenols and aldehydes (Figure 4). Evaluating sample F5B, it is possible to assert that VOCs formation was strongly influenced by the fermentation time. In fact, the sample F5B at 120 days revealed a distinct VOCs profile, displaying a positive correlation with esters, acids, and phenols. Similarly, spontaneous fermentation samples (F5C and F8C) were grouped based on fermentation time, showing a divergent VOCs profile through the fermentation.

### Sensory Data

Table 5 shown results of sensory analysis. Overall, none negative sensation was perceived, as deduced by the low scores attributed by panelist to these descriptors. No statistically significant differences were achieved among samples for hardness, fibrousness, and crunchiness. Among gustatory descriptors, higher scores for acidity were attributed to un-inoculated brine samples at both 5 and 8% of NaCl (F5C and F8C), while higher bitterness score was observed in samples without the addition of the β-glucosidase *L. plantarum* strain (F5C, F5D, F8C, and F8D). Finally, F5B and F8B samples received higher scores for the overall acceptability descriptor.

**Table 5:** Sensory data obtained for the evaluation of fruits in the different treatments assayed.

	**Descriptors**												
	**Abnormal fermentation**							**Gustatory sensations**			**Kinaesthetic sensations**			**Overall acceptability**
**Samples**	**Musty**	**Rancid**	**Cooking effect**	**Soapy**	**Metallic**	**Earthy**	**Winey-vinegary**	**Acidity**	**Saltiness**	**Bitterness**	**Hardness**	**Fibrousness**	**Crunchiness**	
F5A	1.1 ± 0.18^a^	0.6 ± 0.29^a^	0.6 ± 0.22^a^	0.2 ± 0.14^a^	0.6 ± 0.26^a^	1.6 ± 0.12^a^	1.3 ± 0.24^a^	2.7 ± 0.18^a^	5.4 ± 0.11^a^	2.3 ± 0.09^a^	6.4 ± 0.24^b^	2.1 ± 0.06^a^	7.2 ± 0.26^a^	7.5 ± 0.04^a^
F5B	1.1 ± 0.37^a^	0.5 ± 0.43^a^	0.6 ± 0.16^a^	0.3 ± 0.11^a^	0.8 ± 0.21^a^	1.5 ± 0.29^a^	1.2 ± 0.28^a^	2.5 ± 0.26^a^	5.3 ± 0.33^a^	2.0 ± 0.14^a^	6.0 ± 0.21^a^	2.8 ± 0.45^a^	7.5 ± 0.19^a^	9.8 ± 0.41^b^
F5C	1.3 ± 0.21^a^	0.6 ± 0.34^a^	0.8 ± 0.24^a^	0.4 ± 0.21^a^	0.6 ± 0.09^a^	1.6 ± 0.26^a^	1.0 ± 0.31^a^	7.9 ± 0.12^b^	5.4 ± 0.25^a^	6.7 ± 0.24^b^	5.9 ± 0.33^a^	2.2 ± 0.28^a^	7.6 ± 0.21^a^	6.9 ± 0.11^a^
F5D	1.1 ± 0.38^a^	0.5 ± 0.26^a^	0.7 ± 0.26^a^	0.3 ± 0.04^a^	0.7 ± 0.19^a^	1.6 ± 0.61^a^	0.8 ± 0.21^a^	2.9 ± 0.21^a^	5.8 ± 0.34^a^	6.9 ± 0.29^b^	6.3 ± 0.25^a^	2.5 ± 0.36^a^	7.7 ± 0.28^a^	6.7 ± 0.32^a^
F8A	1.1 ± 0.26^a^	0.4 ± 0.22^a^	0.5 ± 0.18^a^	0.3 ± 0.04^a^	0.5 ± 0.24^a^	1.4 ± 0.58^a^	1.2 ± 0.39^a^	2.7 ± 0.61^a^	5.4 ± 0.10^a^	2.5 ± 0.09^a^	6.2 ± 0.18^a^	2.7 ± 0.29^a^	7.6 ± 0.35^a^	7.6 ± 0.09^a^
F8B	1.3 ± 0.24^a^	0.6 ± 0.28^a^	0.8 ± 0.23^a^	0.2 ± 0.35^a^	0.5 ± 0.22^a^	1.4 ± 0.29^a^	0.9 ± 0.15^a^	2.6 ± 0.86^a^	5.6 ± 0.06^a^	2.6 ± 0.32^a^	5.9 ± 0.24^a^	2.1 ± 0.43^a^	7.5 ± 0.32^a^	9.3 ± 0.45^b^
F8C	1.4 ± 0.27^a^	0.4 ± 0.21^a^	0.5 ± 0.26^a^	0.4 ± 0.25^a^	0.7 ± 0.13^a^	1.5 ± 0.54^a^	1.3 ± 0.44^a^	8.8 ± 0.37^c^	5.7 ± 0.21^a^	7.3 ± 0.43^b^	6.2 ± 0.05^a^	2.7 ± 0.51^a^	7.6 ± 0.33^a^	6.4 ± 0.42^a^
F8D	1.4 ± 0.32^a^	0.3 ± 0.35^a^	0.7 ± 0.28^a^	0.4 ± 0.23^a^	0.8 ± 0.31^a^	1.4 ± 0.61^a^	1.3 ± 0.43^a^	2.9 ± 0.53^a^	5.7 ± 0.34^a^	7.2 ± 0.32^b^	6.4 ± 0.09^a^	2.5 ± 0.44^a^	7.8 ± 0.31^a^	6.8 ± 0.38^a^

*a–c: for each descriptor, in the same column, with different superscript letters are significantly different (P < 0.05)*.

## Discussion

A current challenge in the processing technology of table olives is the selection of starter cultures able to fasten and safely drive the fermentation process. In contrast to industrial starter cultures, autochthonous strains, that naturally dominate spontaneous fermentation, tend to have high metabolic capacities, which can beneficially affect the quality of the final product. In addition, it was already established that the microbial dynamics through fermentation is influenced by the technology applied (e.g., salt reduction). One of the most widely employed strategies to reduce sodium content in table olives is the use of NaCl substitutes, which can be added alone or in combination with other salts (Bautista-Gallego et al., 2013a). Few studies have evaluated the possibility to setup low NaCl table olives without any salt replacement. Based on our previously reported data (Randazzo et al., 2017; Pino et al., 2018a), in the present study a wild β-glucosidase positive strain was used both as debittering and as driven agent during olive fermentations at lowered salt content (5%). It is well-known that β-glucosidase enzyme is important for oleuropein hydrolysis and, among lactobacilli, *L. plantarum* species has been successfully used as starter for its strong ability to break the glycosidic bond of oleuropein (Ciafardini et al., 1994; Tataridou and Kotzekidou, 2015) and for its high versatility. Overall, our data revealed that all brine samples reached a pH value ≤ 4.3 and exhibited a good acidification rate, indicating the success of the fermentation and ensuring the microbiological safety of the final product, in accordance to other researches (Corsetti et al., 2012; Martorana et al., 2017). In particular, samples inoculated with the β-glucosidase positive strain exhibited a more pronounced reduction of the fermentation time, with a higher content of hydroxytyrosol, tyrosol, and verbascoside compounds from 30 days of fermentation than un-inoculated ones, according to other studies (Romero et al., 2004; Ben Othman et al., 2009; Pistarino et al., 2013; Kaltsa et al., 2015). In addition, the autochthonous strain exhibited a better adaptation/growth rate in brine samples at 5% of salt. It is well-established that autochthonous strains are generally more adapted to harsh conditions of raw material than allochthonous ones, and, therefore, to dominate the microbiota, driving the fermentation and counteracting spoilage microorganisms (Di Cagno et al., 2008; Bevilacqua et al., 2015). Microbiological data indicated a significant reduction of *Enterobacteriaceae* starting from 30 days of fermentation, with an improvement of the safety of the final product, as previously reported (Pino et al., 2018a). It is interesting to point out that results revealed a high occurrence of yeasts as part of table olive natural microbiota, coexisting with LAB during the whole fermentation process (Arroyo-López et al., 2008, 2012a,c), which could be related to the geographic area, and cultivars (Bleve et al., 2014). Our data registered an unusual yeast count at the end of the process, higher than Spanish-style and Sicilian-style table olives, which generally reached value of 4-5- log CFU/ml. This could be linked to the processing technology applied in the present study. Yeasts favor bacteria growth, enhancing lactic acid production to inhibit spoilage microorganisms and affect flavor and texture of the final products (Arroyo-López et al., 2008, 2012b; Bevilacqua et al., 2013). As reported in a recent review, *Candida boidinii, Debaryomyces hansenii*, and *Pichia membranifaciens* were revealed as the most geographically diffused species (Campus et al., 2018). Evaluating yeast behavior, although it is noteworthy that yeast development is related to high salt level and phenolic compounds or low pH. In the present study, *W. anomalus*, and *C. boidinii* were the species mainly detected in brines processed at 5% of salt, whereas *C. diddensae* and *M. guilliermondii* were mainly revealed in brines at 8% NaCl. Several studies reported strong β-glucosidase activity for *W. anomalus* species (Bautista-Gallego et al., 2011b; Arroyo-López et al., 2012c; Romero-Gil et al., 2013; Bonatsou et al., 2015), and strong lipase and esterase activities for *C. boidinii* species, which positive impacts to fruity and olive flavor (Hernández et al., 2007; Bautista-Gallego et al., 2011b; Arroyo-López et al., 2012c; Pereira et al., 2015). In contrast to previously published data (Hurtado et al., 2008; Bautista-Gallego et al., 2011b), *C. diddensae*, which is generally associated to the early stage of fermentation, was detected at the highest frequency at 120 days. Focusing on LAB population, *L. plantarum, L. pentosus*, and *L. paracasei* were the main species found in all brine samples, confirming both their key role in table olive fermentation and biofilm formation with yeasts. In addition, a high survivability of the potential probiotic *L. paracasei* N24 strain was depicted in the final products, mainly in samples at 5% of NaCl. This evidence confirms its suitability to growth in harsh environment, such as brines, and that table olives are able to support probiotic survival (Lavermicocca et al., 2005; Rodríguez-Gómez et al., 2017; Pino et al., 2018a). In fact, nutrients and prebiotics released into the brines favor the biofilm formation, protecting bacteria from acidic environment and enhancing their passage through human gastrointestinal (GI) tract (De Bellis et al., 2010; Ranadheera et al., 2010; Arroyo-López et al., 2012a; Blana et al., 2014; Rodríguez-Gómez et al., 2014a,b, 2017; Grounta et al., 2015). It is interesting to point out that the addition of the potential probiotic strain at 60 days of fermentation in brine samples processed at low salt content and with starter, significantly modified the VOCs pattern. In particular, compounds responsible for floral and fruity notes, such as phenylethyl alcohol and methyl 2-methylbutanoate, highly increased, while ethanol and isoamyl-alcohol significantly deceased compared to un-inoculated samples. The high content of alcohols in un-inoculated brine samples could be related to yeast metabolic activities (Bleve et al., 2014; Randazzo et al., 2017). This evidence was in accordance to sensory data since panelists attributed the higher score to the bitterness and acid descriptors in un-inoculated samples. Finally, data obtained from correlation between VOCs and brine samples differently treated revealed that the VOCs formation was mainly influenced by the starter and/or probiotic addition instead of salt content.

## Conclusion

The effects of a sequential inoculum of β-glucosidase positive and potential probiotic strains on the fermentation of Sicilian table olives were investigated. Remarkably, results demonstrate that the technology applied, based on the sequential inoculum and the brines fermentation at low salt content, without any salt replacement, did not increase the risk of microbial spoilage, nor the overgrowth of foodborne pathogens. Indeed, the composition and the dynamics of brine microbiota, mainly constituted by LAB and yeasts consortium, significantly affected the composition of the VOCs and the sensorial traits of the final products, which were confirmed by a panel of trained assessors. Hence, the results of the present study are promising, suggesting the possibility to formulate table olives with reduced salt content.

## Author Contributions

AP, FR, AT, AV, and LS performed the experiments and analyzed the data. AP, JB-G, and CR wrote the manuscript. CR, JB-G, and CC designed the study. CR, JB-G, and FA-L contributed to data interpretation. All authors revised the manuscript.

### Conflict of Interest Statement

The authors declare that the research was conducted in the absence of any commercial or financial relationships that could be construed as a potential conflict of interest.
