# Supplementary material for: Effect of Sequential Inoculum of Beta-Glucosidase Positive and Probiotic Strains on Brine Fermentation to Obtain Low Salt Sicilian Table Olives
Source: Front Microbiol. 2019 Feb 8;10:174. doi: 10.3389/fmicb.2019.00174 (PMC6376858; doi:10.3389/fmicb.2019.00174)
Supplement: Supplementary file 1 [file Table_1.DOCX]

Supplementary Material

Effect of sequential inoculum of beta-glucosidase positive and probiotic strains on brine fermentation to obtain low salt Sicilian table olives

**Alessandra Pino^1^, Amanda Vaccalluzzo^1^, Lisa Solieri^2^, Flora V. Romeo^3^, Aldo Todaro^4^, Cinzia Caggia^1^, Francisco Noé Arroyo-López^5^, Joaquin Bautista-Gallego^5^, Cinzia L. Randazzo*^1^**

**Correspondence:** Corresponding Author: cranda@unict.it

# Supplementary Figure

**Supplementary Figure 1.** Representative electrophoresis gel picture reporting GTG_5_-based band patterns of the inoculated probiotic strain N24 and 10 isolates. Isolates code are according to brine samples and were reported on the top. Blue and red colours are in agreement with color code of clusters A and B/C/D in Figure 3.
