# Supplementary figures and images for: Effect of Sequential Inoculum of Beta-Glucosidase Positive and Probiotic Strains on Brine Fermentation to Obtain Low Salt Sicilian Table Olives
Source: Front Microbiol. 2019 Feb 8;10:174. doi: 10.3389/fmicb.2019.00174 (PMC6376858; doi:10.3389/fmicb.2019.00174)

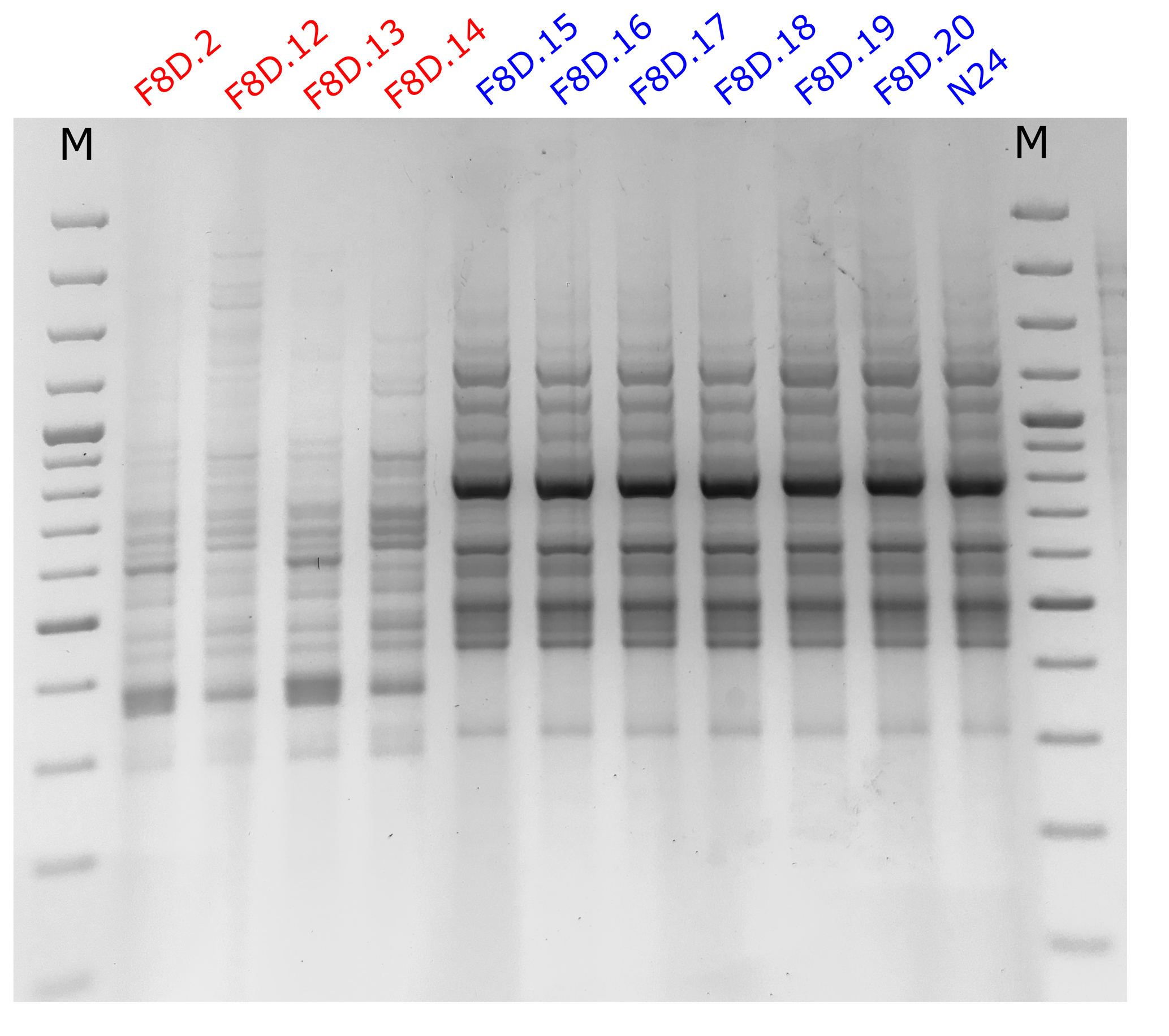

Supplement: Supplementary file 2 [file Image_1.TIF]
